# *Trichoderma* gets by with a little help from *Streptomyces*: fungal–bacterial symbiosis in plant growth promotion

**DOI:** 10.1093/jxb/erae439

**Published:** 2024-12-03

**Authors:** Tessa E Reid, Miriam L Gifford

**Affiliations:** Sustainable Soils and Crops, Rothamsted Research, Harpenden, Hertfordshire AL5 2JQ, UK; School of Life Sciences and The Zeeman Institute for Systems Biology & Infectious Disease Epidemiology Research, The University of Warwick, Gibbet Hill Road, Coventry CV4 7AL, UK

**Keywords:** Bioinoculants, helper microbes, plant growth promotion, rhizosphere, sorghum, sustainable agriculture, *Trichoderma*

## Abstract

This article comments on:

**Kabir AH, Thapa A, Hasan R, Parvej R.** 2024. Local signal from *Trichoderma afroharzianum* T22 induces host transcriptome and endophytic microbiome leading to growth promotion in sorghum. Journal of Experimental Botany **75**, https://doi.org/10.1093/jxb/erae340.

This article comments on:

**Kabir AH, Thapa A, Hasan R, Parvej R.** 2024. Local signal from *Trichoderma afroharzianum* T22 induces host transcriptome and endophytic microbiome leading to growth promotion in sorghum. Journal of Experimental Botany **75**, https://doi.org/10.1093/jxb/erae340.

**Plant–microbe interactions are crucial for plant health and agricultural sustainability. *Trichoderma* species are common soil and root fungi that have been widely studied due to their capacity to produce antibiotics, parasitize other fungi, and compete with deleterious plant microorganisms, but they are also emerging as promising plant growth promoters. Kabir *et al*. (2024) revealed that *Trichoderma afroharzianum* T22 promotes sorghum growth through local signalling and by modulating the plant transcriptome and microbiome. The study showed how the intricate interplay among *T. afroharzianum*, helper microbes such as *Streptomyces*, and the sorghum host drives symbiotic growth promotion**.

It is hard not to notice the concerning impacts of climate change daily. One major accelerator has been the intensification of agriculture, propelled by the widespread and indiscriminate use of fungicides, herbicides, and inorganic fertilizers, which has affected soil health and provoked biodiversity degradation and acidification. To sustainably transform the agricultural system, one key direction is to explore organic bio-based pesticide and fertilization techniques that enhance soil fertility and health. A method with significant potential is the formulation of synthetic microbial inoculants that can be applied to soils to increase crop yields whilst also maintaining soil health. An impressive example is *Trichoderma*, an ascomycete fungal genus containing several species of interest to agriculture, as biocontrol agents of phytopathogens and plant growth promoters. It is one of the most widely used biocontrol agents in agriculture, with *Trichoderma*-based products commercially available and utilized globally (Woo *et al*., 2014; Tyśkiewicz *et al*., 2022). Like other filamentous fungi, *Trichoderma* colonizes the root system (rhizosphere and roots), producing anti-microbial and biostimulating compounds while interacting with other rhizosphere microbes (Li *et al*., 2020). The efficacy and reliability of *Trichoderma* products depend on their competitiveness in the root system, which can be enhanced by combining them with other microorganisms, known as helper microbes, that support the positive effects of *Trichoderma* (Box 1).

## Unravelling the mechanism by which *Trichoderma* promotes plant growth

*Trichoderma* spp. influence both bacterial and fungal communities in the rhizosphere of various plants (reviewed in Asghar *et al*., 2024). The effects of *Trichoderma* on the rhizosphere microbiome are highly plant specific, suggesting that signals from the root influence the resident microbial responses. Kabir *et al*. (2024) demonstrated that the commercial inoculum (Trianum-P) of *T. afroharzianum* strain T22 significantly increased above- and below-ground sorghum biomass as well as nutrient uptake. RNA sequencing of sorghum roots colonized by *T. afroharzianum* showed up-regulation of genes involved in nutrient transport, auxin signalling, and pathogen defence. This transcriptional reprogramming is likely to underpin the observed growth promotion. The authors also analysed changes in the root microbiome, finding that *T. afroharzianum* enriches potentially beneficial microbes such as *Streptomyces* and *Penicillium*, while reducing pathogenic *Fusarium* fungi. The interaction between *Trichoderma* and *Fusarium* is complex; *Trichoderma* inoculation also suppressed *Fusarium* in cucumber but increased its abundance in cabbage and black pepper (Umadevi *et al*., 2018; Hang *et al*., 2022; Wang *et al*., 2023), suggesting that *Trichoderma* can have either a positive or a negative interaction with *Fusarium*.

*Trichoderma* is emerging as a promising microbial inoculant for enhancing biofertilization, biocontrol, and abiotic stress tolerance in sorghum. In this study, inoculation of *T. afroharzianum* T22 led to increased nutrient content (Fe, Zn, Mn, Mg, and Ca) in sorghum shoots under controlled pot conditions. These findings align with broader research demonstrating the benefit of *Trichoderma* in sorghum cultivation. Inoculation with *Trichoderma harzianum* increased yield and sugar content in field-grown sorghum under salinity stress (Wei *et al*., 2023). It also improved physiological traits, Fe levels, and redox status in sorghum grown in iron-deficient soils (Kabir and Bennetzen, 2024). Furthermore, *Trichoderma* treatment reduced the incidence of sorghum anthracnose (caused by *Colletotrichum graminicola*) in both pot and field trials (Manzar *et al*., 2021). These studies collectively highlight the diverse benefits of *Trichoderma* across various growth conditions and stress factors in sorghum production.

Differentiating between local signalling pathways that act at the cell level and systemic signalling pathways that enable communication across different tissues and plant organs can help to elucidate physiological mechanisms. The authors used a split-root experiment and revealed that *T. afroharzianum* must be present throughout the root system in order to improve sorghum yield. *Trichoderma* presence only increased the root biomass of the compartment it was in but was required in both compartments to confer above-ground plant growth-promoting effects, suggesting that *Trichoderma* promoted growth through localized signalling rather than systemic plant responses (Kabir *et al*., 2024). In comparison, a split-root experiment with garden pea showed that the presence of *T. afroharzianum* in the rhizosphere, even when split across two compartments, was sufficient for overall plant (root/shoot) growth promotion, suggesting that *Trichoderma* can act both locally and systemically, depending on the plant genetic response and environmental conditions (Thapa *et al*., 2024, Preprint).

Synergies between *Trichoderma* and microbes can cause more benefits than the sum of their parts, and this makes them a promising alternative for managing crops and controlling diseases or pests in modern agriculture (Poveda and Eugui, 2022). To test whether the growth promotion mediated by *T. afroharzianum* could be influenced by the presence or absence of additional microbes, Kabir *et al*. (2024) conducted an experiment with synthetic microbial consortia based on the enriched taxa from the bacterial and fungal community analysis. Interestingly, they found that *Streptomyces griseus* acted as a helper microbe, enhancing the growth-promoting effects of *T. afroharzianum* when co-inoculated (Box 1).

Based on these and previous findings, we can begin to build a roadmap from *Trichoderma* inoculation to plant growth promotion. When *Trichoderma* is applied to soils, it establishes its fungal network through the growth of filaments and proceeds to the rhizosphere to colonize the root system. In the rhizosphere, *Trichoderma* begins to exchange signalling molecules with the plant using its suite of secondary metabolites which stimulate plant gene expression (Contreras-Cornejo *et al*., 2016). In the early stages of the interaction, auxin-like metabolites and proteinaceous compounds released by *Trichoderma* are perceived by the roots, altering multiple hormonal mechanisms that control plant growth and development under ‘normal’ or stress conditions. *Trichoderma*–bacterial and *Trichoderma*–fungal interactions influence the abundance of microbes in the rhizosphere. Consequently, when the root system is colonized, the association is potentiated, providing protection in this zone against pathogenic microorganisms. In addition, a robust root system is developed, improving nutrient and water uptake.

## One size does not fit all: crop-specific *Trichoderma* amendments

As climate change threatens crop yields globally, leveraging beneficial microbes to enhance plant resilience and productivity will be crucial for food security. The multifaceted way with which *Trichoderma* interacts with its host plant species means that tailoring inoculation to the crop type remains challenging (Shahriar *et al*., 2022). *Trichoderma* has been shown to interact with >70 plant species (Contreras-Cornejo *et al*., 2016). The pipeline with which Kabir *et al*. (2024) investigated *Trichoderma* interactions proved to be an excellent way to decipher the mechanisms and identify helper microbes that could be co-inoculated with *Trichoderma* for improved plant growth (Fig. 2). This same pipeline could also be applied to determine the mechanism with which microbial inoculants cause disease resistance and abiotic tolerance in different crops, thus allowing screening of multiple crop species. The synergistic effects of *Trichoderma* and helper microbes suggest that multi-species microbial consortia may be more effective biofertilizers than single strains.

**Fig. 1.**
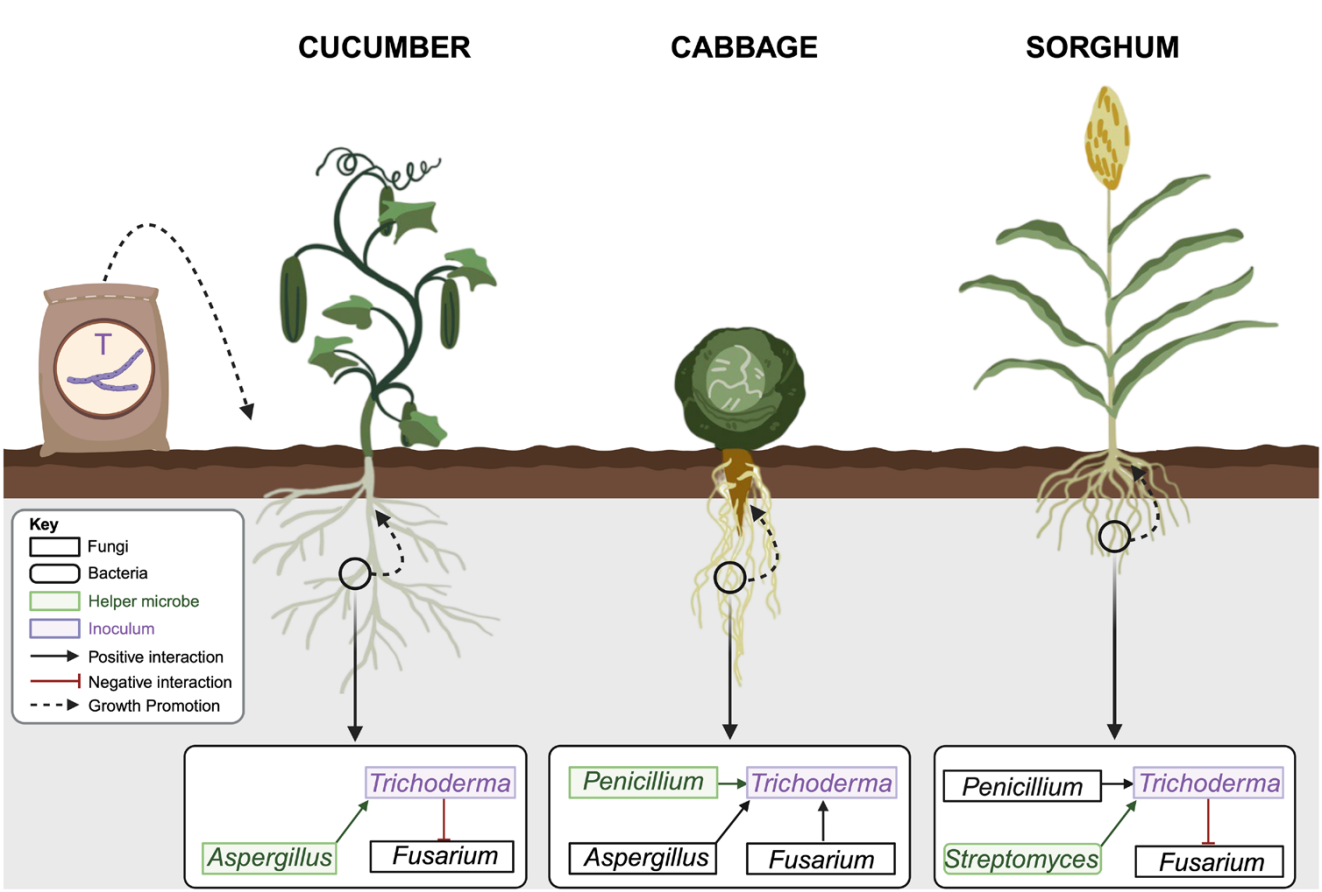
Interaction of *Trichoderma* and helper microbes. *Trichoderma* inoculation of soils stimulates different root microbiome responses, depending on the crop type, with different helper microbes. In cucumber, it increases *Aspergillus* growth, which in turn stimulates the growth-promoting effects from *Trichoderma*, while suppressing *Fusarium* growth (Hang *et al*., 2022). In cabbage, *Aspergillus*, *Fusarium*, and *Penicillium* growth is stimulated, with *Penicillium* increasing the growth promotion effects from *Trichoderma* (Wang *et al*., 2023). In sorghum, *Trichoderma* increases the growth of *Penicillium* and *Streptomyces*, with *Streptomyces* increasing the growth-promoting effects from *Trichoderma*, while repressing *Fusarium* growth (Kabir *et al*., 2024). Created with BioRender.com.

**Fig. 2.**
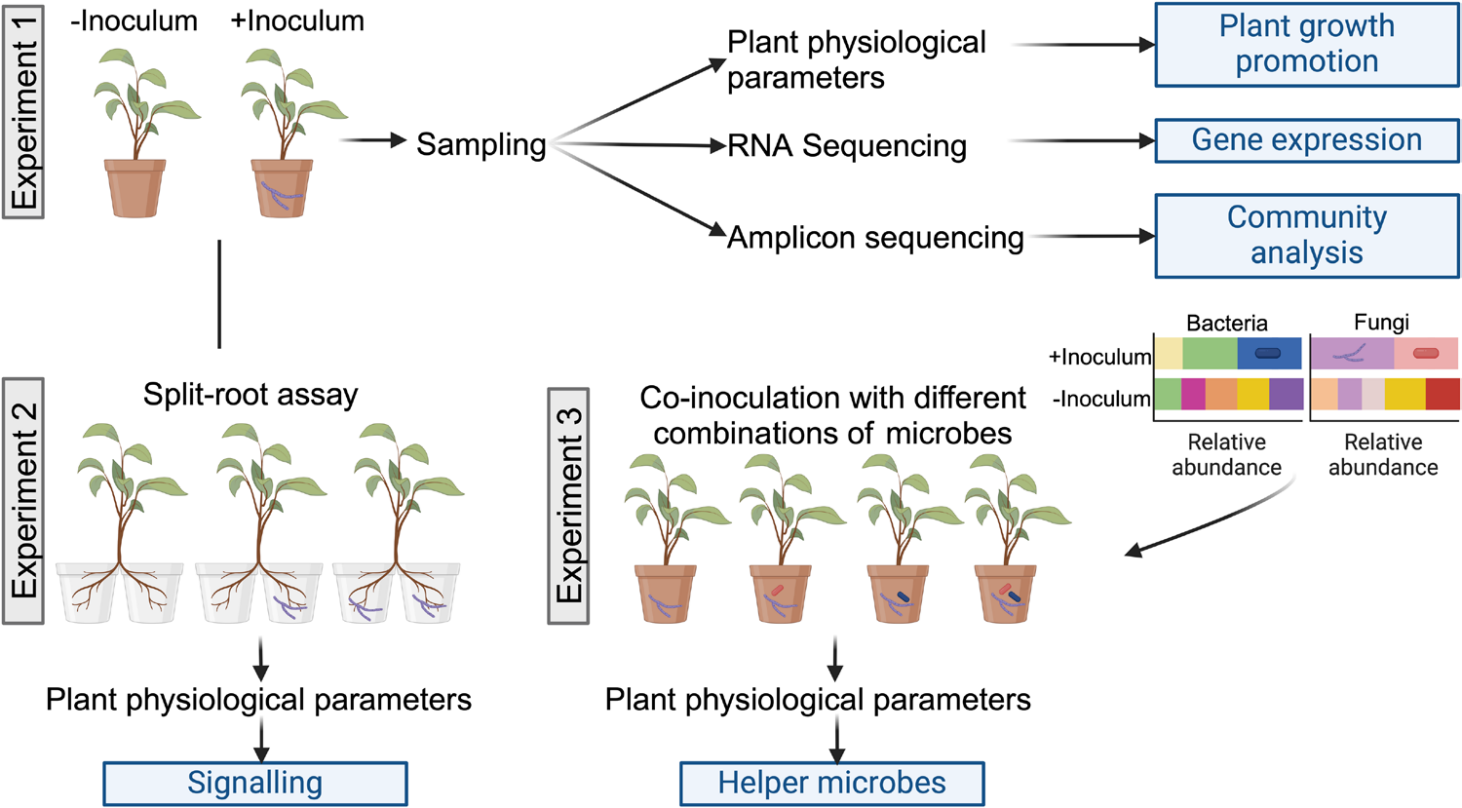
Experimental pipeline to determine the mechanism of plant growth promotion by microbial inoculants. Schematic diagram depicting three complementary experiments employed by Kabir *et al*. (2024). Experiment 1: inoculation (+Inoculum) versus no inoculation (–Inoculum) of a plant followed by sampling (roots and aerial plant) and determination of plant physiological parameters, RNA sequencing of plant tissue (roots/shoots), and amplicon sequencing to determine any growth promotion (increased biomass, nutrient uptake etc.), differences in gene expression, and fungal and bacterial community changes caused by the inoculum, respectively. Experiment 2: a split-root assay to determine if plant growth promotion occurs when the inoculum is present exclusively in both compartments (local signalling) or in one compartment (systemic signalling). Experiment 3: community analysis from experiment 1 guides the combination of co-inoculants based on taxa that had a significantly higher abundance with inoculation. Co-inoculations which improve plant growth parameters more than the single inoculum are likely to be helper microbes. Created with BioRender.com.

The status of *Trichoderma* as a commercially available bioinoculant has the advantage that it is already approved for agricultural use and widely applied. As demonstrated by Kabir *et al*. (2024), *Trichoderma* acts locally on sorghum, which suggests that a homogenous distribution of *Trichoderma* inoculant applied across the soil system is required to fully exploit the beneficial effects from these fungi. By shedding light on the molecular and ecological mechanisms underlying this symbiosis, new avenues are opened up for developing more effective and sustainable agricultural biotechnologies.

Box 1. **Helper microbes improve plant growth promotion of *Trichoderma***

Helper microbes were first described in respect to bacteria that have the ability to promote the establishment of arbuscular mycorrhizal fungi (AMF) root symbiosis, termed mycorrhiza helper bacteria (MHB) (Garbaye, 1994). MHB can impact the functions of an already established AMF symbiosis or stimulate the establishment of the fungal symbionts on the host plants (Frey-Klett *et al*., 2007).

Whilst *Trichoderma* helper microbes have rarely been described, synergies with plant growth-promoting bacteria have been reported to potentiate the biocontrol efficiency of plant diseases (reviewed in Guzmán-Guzmán *et al*., 2024). Here, previous studies investigating the interactions of *Trichoderma* in the rhizosphere substantiate the concept of *Trichoderma* helper microbes (Fig. 1). Community analysis in the sorghum rhizosphere showed an increase in the abundance of *Penicillium* and *Streptomyces*, with co-inoculation experiments showing that only the presence of *Streptomyces* was essential for growth promotion (Kabir *et al*., 2024). In the cucumber rhizosphere, the abundance of *Aspergillus* increased with *Trichoderma* application; co-inoculation experiments showed that the combination of these microbes improved the growth of cucumber when compared with single inoculations (Hang *et al*., 2022). In the ground vegetable cabbage, plant growth promotion by *Trichoderma guizhouense* was enhanced by co-inoculation with *Aspergillus*, *Fusarium*, or *Penicillium* individually, with *Penicillium* showing the strongest growth promotion (Wang *et al*., 2023). An emerging theme is that co-inoculation experiments are essential in identifying specific helper microbes. While community analysis determines shifts in taxa abundance, it does not yet answer the question of whether *Trichoderma* requires these microbes to promote plant growth. These findings collectively emphasize the complexity of *Trichoderma*-mediated microbiome modulation and underscore the need for crop-specific investigations to harness these interactions for sustainable agriculture.
